# The NF-κB Pathway: Modulation by *Entamoeba histolytica* and Other Protozoan Parasites

**DOI:** 10.3389/fcimb.2021.748404

**Published:** 2021-09-14

**Authors:** Attinder Chadha, Kris Chadee

**Affiliations:** Departments of Microbiology, Immunology, and Infectious Diseases, Cumming School of Medicine, Snyder Institute for Chronic Diseases, University of Calgary, Calgary, AB, Canada

**Keywords:** entamoeba histolytica, macrophage, NF-κB – nuclear factor kappa B, innate immunity, cytokine

## Abstract

Protozoan parasites have led to worldwide devastation because of their ability to cause infectious diseases. They have evolved as successful pathogens in part because of their remarkable and sophisticated ways to evade innate host defenses. This holds true for both intracellular and extracellular parasites that deploy multiple strategies to circumvent innate host defenses for their survival. The different strategies protozoan parasites use include hijacking the host cellular signaling pathways and transcription factors. In particular, the nuclear factor-κB (NF-κB) pathway seems to be an attractive target for different pathogens owing to their central role in regulating prompt innate immune responses in host defense. NF-κB is a ubiquitous transcription factor that plays an indispensable role not only in regulating immediate immune responses against invading pathogens but is also a critical regulator of cell proliferation and survival. The major immunomodulatory components include parasite surface and secreted proteins/enzymes and stimulation of host cells intracellular pathways and inflammatory caspases that directly or indirectly interfere with the NF-κB pathway to thwart immune responses that are directed for containment and/or elimination of the pathogen. To showcase how protozoan parasites exploits the NF-κB signaling pathway, this review highlights recent advances from *Entamoeba histolytica* and other protozoan parasites in contact with host cells that induce outside-in and inside-out signaling to modulate NF-κB in disease pathogenesis and survival in the host.

## Introduction

Protozoan parasites have been a major concern due to their ability to cause considerable mortality and morbidity in both humans and animals worldwide (Dorny et al., 2009; Dixon et al., 2011; Fletcher et al., 2012; Kelly, 2013). They are responsible for affecting more than 500 million people across the globe (Monzote and Siddiq, 2011). Although parasitic infection and death are a major cause of concern in developing countries, they are also responsible for causing significant illness in developed countries (Fletcher et al., 2012). The burden of human protozoan parasitic infections has been aggravated because of the lack of a licensed vaccine against any of the diseases these parasites cause. Moreover, prophylaxis and treatment are dependent on drugs, which are rendered ineffective in many cases due to the emergence of drug resistance warranting the search for replacements (Andrews et al., 2014).

Protozoan parasites are unicellular eukaryotic that either reside extracellularly or intracellularly in host cells. They have evolved as successful pathogens due to their remarkable ability to evade immune responses allowing them to escape adaptive humoral and cellular immunity (Sacks and Sher, 2002). For instance, *Toxoplasma gondii* (Lima and Lodoen, 2019), *Leishmania* (Gupta et al., 2013) and *Trypanosoma cruzi* (Cardoso et al., 2016) evade humoral antibody response by adopting an intracellular lifestyle, while antigenic variations, in the case of extracellular pathogens such as *Giardia* (Prucca and Lujan, 2009), African trypanosomes (Horn, 2014), and malarial parasites (Kyes et al., 2001) that express their antigens on the surface of red blood cells, help them overcome immune destruction.

Although pathogens deploy different strategies for immune subversion, modulation of the NF-κB pathway critical for generating an immune response seems to be a crucial target (Tato and Hunter, 2002). While the NF-κB pathway is critical for mounting an immune response, pathogens have devised multiple ways to thwart this pathway to their advantage including, bacteria (Le Negrate, 2012), viruses (Santoro et al., 2003), and protozoan parasites (Heussler et al., 2001). Pathogens or their components have a remarkable ability for interfering with the NF-κB pathway at multiple levels which includes, membrane-bound receptors to downstream signaling molecules of the pathway. Host-pathogen interaction can have multiple outcomes, but pathogens that circumvent signaling pathways seem to establish a successful niche for their replication and to cause disease. Both extracellular protozoan parasites *via* outside-in-signaling and intracellular protozoan parasites *via* inside-out-signaling have devised unique ways to overcome innate defense barriers by modulating the NF-κB pathway at multiple levels. To understand the complex interaction whereby protozoan parasite interacts with the NF-κB pathway, this review will focus on recent findings on modulation of NF-κB signaling with the extracellular parasite *Entamoeba histolytica* (*Eh*) and the intracellular parasite, *T. gondii*.

## The NF-κB Pathway

**Figure 1:**
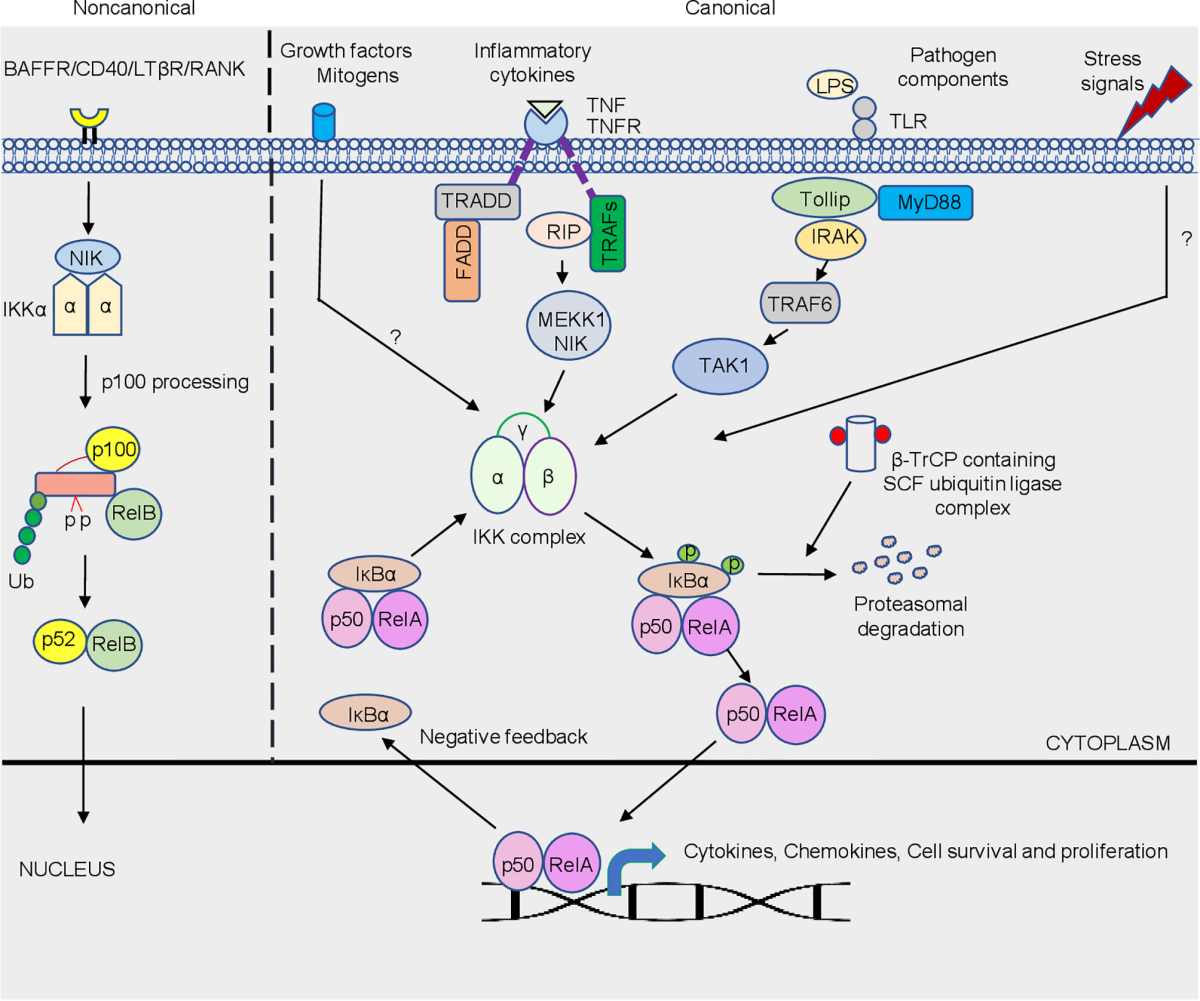
Schematic representation of the canonical and non-canonical NF-κB signaling pathway. The canonical pathway is activated by a plethora of trigger/stimuli that includes different pathogens, stress signals, growth factors and inflammatory cytokine exposure which converges on the IKK complex. Activation of the NF-κB is tightly regulated due to the sequestration of the complex by IκBα in the cytosol. Phosphorylation of IκBα *via* IKK is a signal for its degradation, which is mediated by β-TrCP containing SCF-ubiquitin ligase complex. Freed dimers subsequently translocate to the nucleus where they bind to κB elements that controls the transcriptions of a variety of genes, which includes genes responsible for cytokine, chemokines, cell survival and proliferation. The non-canonical NF-κB pathway is dependent on the phosphorylation-induced p100 processing triggered by signaling from a subset of TNFR members. This pathway is reliant on NIK and IKKα, but not on the trimeric IKK complex, and mediates the activation of RelB/p52 complex. The detailed pathway is described in text.

NF-κB activation is a rapid event that occurs within minutes upon any trigger or stimulation that regulates a myriad of genes in host cells and does not require protein synthesis which makes this pathway an attractive target for invading pathogens (Santoro et al., 2003). NF-κB regulates diverse cellular function (**Figure 1** **)** which includes, promoting inflammation, an early response to pathogen that plays an indispensable role in cell survival and proliferation (Karin et al., 2002; Li and Verma, 2002). It comprises of dimeric transcription factors belonging to the Rel family. Five Rel proteins belonging to two different classes have been identified in mammalian cells (Ghosh et al., 1998; Santoro et al., 2003). c-Rel, RelA (p65) and RelB belong to one class, that are synthesized as matured form, and contain an N-terminal Rel homology domain (RHD) responsible for dimerization and DNA binding, and C-terminus that possess transcription modulating domains (Verma et al., 1995; Santoro et al., 2003; Gilmore, 2006). Another class comprise of an N-terminal RHD and a C-terminal ankyrin repeat domain-containing p105 and p100 precursor proteins that require ubiquitin-dependent processing at the C-terminus. Thus, the mature DNA-binding proteins of this class contain N-terminal RHD but lack C-terminus transcription modulating activity (Santoro et al., 2003; Gilmore, 2006). NF-κB, whose predominant form p50 and RelA subunits, remains inactive in the cytoplasm because of its association with inhibitor proteins known as inhibitors of NF-κB (IκBs), including IκBα, IκBβ and IκBϵ (Verma et al., 1995; Ghosh et al., 1998; Santoro et al., 2003). The mechanism of NF-κB activation is tightly regulated. Different stimuli or trigger, including bacterial, viral, and protozoan parasite infections may culminate in phosphorylation of IκB proteins, leading to ubiquitination and proteasomal degradation of phosphorylated IκB proteins (**Figure 1**). The degradation of IκB sets free NF-κB that translocates to the nucleus and binds to DNA to control the transcription of different genes including, cytokines, chemokines, antimicrobial peptides, anti-apoptotic proteins, and stress-response proteins. The NF-κB pathway is activated by signaling through multiple receptors on the cell membrane. Amongst the different sensors, TLRs (Toll-like receptor) are important pathogen recognition receptors (PRR) that bind bacterial products and LPS (lipopolysaccharide) to initiate downstream signaling cascade culminating into NF-κB activation. Binding of bacterial products/LPS to TLRs initiates downstream signaling leading to the recruitment of MyD88 (myeloid differentiation primary response gene 88), a death-domain containing adaptor protein and Toll-interacting protein Tollip (Silverman and Maniatis, 2001). The pro-inflammatory cytokine TNF (tumor necrosis factor)-α signals *via* the NF-κB pathway. Cognate binding of TNF-α to type 1 TNF-α receptor (TNFR1) recruits the adaptor protein TNFR-associated death domain (TRADD) that acts as a docking site for the receptor interacting protein RIP and TNFR-associated factor TRAF2 that initiates downstream signaling (Chen and Goeddel, 2002). Further, downstream are MAP3K- related kinase which are thought to link receptor-complexes and stimulate an IκB kinase (IKK) complex. TRADD also binds to Fas-associated death domain (FADD) that initiate a protease cascade culminating into apoptosis (Baud and Karin, 2001). Activation of the NF-κB pathway (**Figure 1**) by different stimuli involves distinct scaffolding or signaling proteins, which, in addition to those mentioned above, include mitogen-activated protein kinase/extracellular signal-regulated kinase kinase 1(MEKK1), TNFR-associated factors (TRAFs), protein kinase C (PKC), transforming growth factor-β (TGF-β)-activated kinase (TAK1), NF-κB-inducing kinase (NIK), interleukin (IL)-1-receptor-associated kinases (IRAKs), double-stranded (ds) RNA-dependent protein kinase (PKR) and several others (Silverman and Maniatis, 2001). Most of the above-mentioned proteins execute its effect by acting on another important downstream protein complex, the IκB kinase (IKK) signalosome complex that plays an indispensable role in NF-κB activation (Israël, 2000).

The IKK signalosome complex is a multi-subunit complex comprising of three distinctive subunits IKK-α, IKK-β, and IKK-γ (**Figure 1**). IKK-α and IKK-β form the catalytic center of the complex that exist either as a homo- or heterodimers, and with IKK-γ or NEMO (NF-κB essential modulator) forms the regulatory subunit, that acts as a docking site for the other signaling protein or IKK kinase (Rothwarf et al., 1998; Israël, 2000; Santoro et al., 2003). Integrity of IKK-γ is required for NF-κB activation. The mechanism of NF-κB activation is well orchestrated by serine phosphorylation of IKK-β subunit that is mediated by upstream kinases or through trans autophosphorylation of IKK subunits. Aautophosphorylation of IKK-β at the C-terminal serine cluster prevents prolonged NF-κB activation, thus acting as a negative feedback regulation (Delhase et al., 1999). The phosphorylation of IκB at N-terminal Ser 32 and Ser 36 (Karin and Ben-Neriah, 2000), mediated by IKK, leads to proteasomal degradation of the inhibitory subunit by 26S proteasome, resulting in NF-κB activation. βeta-transducin repeat- containing protein (β-TrCP) containing SCF (Skp1, Cdc53/cullin, and F box protein) ubiquitin ligase mediates the ubiquitination of phosphorylated IκB at Lys21 and Lys22 (Liang et al., 2004). In general, bacterial and viral infections triggered NF-κB activation is mediated by IKK-β. In contrast, a unique regulatory mechanism of the NF-κB pathway *via* the non-canonical arm predominantly targets activation of RelB/p52 subunit (Senftleben et al., 2001). Unlike the canonical pathway that responds to signals elicited by diverse receptors, the non-canonical pathway is targeted by a specific set of receptors (Sun and Harhaj, 2006). The best-characterized non-canonical NF-κB receptors include a subset of the TNFR superfamily members, including B-cell-activating factor belonging to the TNF family receptor (BAFFR; Claudio et al., 2002), lymphotoxin β-receptor (LTβR; (Dejardin et al., 2002), receptor activator for NF-κB (RANK; (Novack et al., 2003) and CD40 (Coope et al., 2002). In resting cells, RelB associates with NF-κB2 p100 polypeptide in the cytoplasm whose C-terminal ankyrin repeat undergoes degradation upon stimulation, releasing RelB-p52 dimers that translocate to the nucleus (Senftleben et al., 2001; **Figure 1**). Activation of this process is mediated by the IKK-α subunit, unlike the canonical NF-κB pathway which is primarily mediated by IKK-β. NIK is a central signaling component of the non-canonical pathway, which integrates signals from a subset of TNF receptor family members and activates a downstream kinase, IKKα, for triggering phosphorylation of p100 and its processing (Sun, 2011). Following activation, NF-κB translocates to the nucleus where it binds to DNA consensus sequence 5’-GGGACTTTCC-3’ (κB elements; **Figure 1**). NF-κB transcriptional activity is greatly enhanced by the phosphorylation of RelA by protein kinase A (PKA) that facilitates its association with the transcriptional coactivator CBP/p300 (Zhong et al., 1998). Importantly, acetylation of NF-κB was described as an additional regulatory mechanism for the activity of NF-κB (Chen et al., 2001).

## NF-κB Regulation During *Entamoeba histolytica* Infection

**Figure 2:**
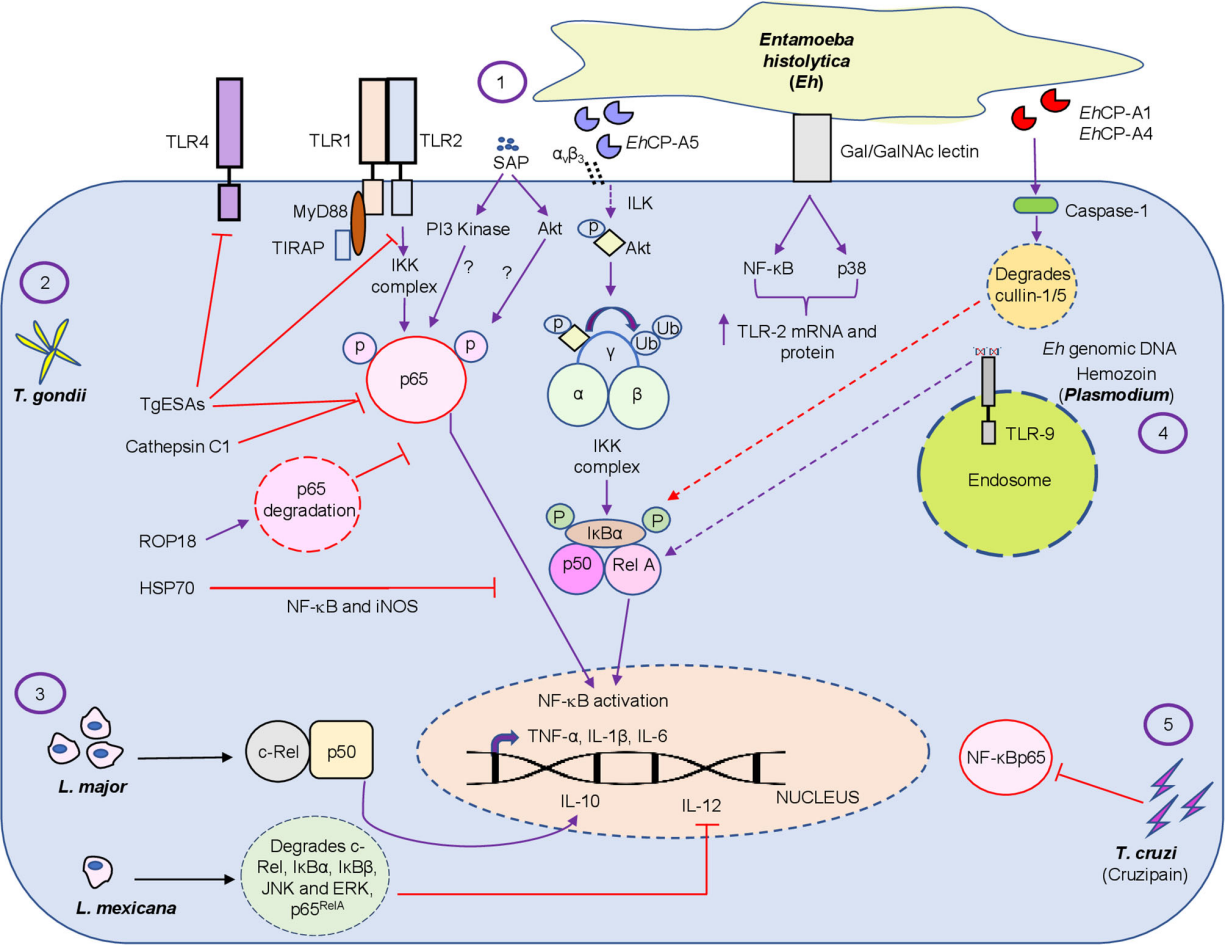
Diagrammatic representation of the intriguing relationship between protozoan parasite virulence factors and the NF-κB pathway. The figure represents the regulation of the NF-κB pathway by the extracellular protozoan parasite *Eh* and its virulence factors, which includes SAP, *Eh*CP-A1, *Eh*CP -A4 and *Eh* genomic DNA (1) and by different intracellular protozoan parasites *T. gondii virulence* factors, namely, TgESAs, Cathepsin C1, ROP18 and HSP70 (2), *L. major/Mexicana* live infection (3), *Plasmodium* pathogenic component hemozoin (4) and *T. cruzi* secreted lysosomal peptidase cruzipain (5) at different levels by modulating the inflammatory response during host-pathogen interaction. Virulence factors and live infection modulates NF-κB signaling at multiple levels. Both intracellular and extracellular protozoan parasites differentially modulate the NF-κB pathway. Note, purple color arrows indicate activation/promotion while red color arrows depict inhibition. The detailed mechanisms of action of the virulence factors are described in the text.

*E. histolytica* (*Eh*) is an extracellular protozoan parasite and the causative agent of the disease amebiasis. *Eh* infects ~10% of the world population leading to 100,000 deaths/year (Stanley Jr, 2003). Though the disease is a concern worldwide, it is more prevalent in developing countries due to poor sanitation and nutrition (Mahmud et al., 2013). Although multiple factors contribute to disease pathogenesis, it is primarily determined by the efficacy and quality of the host immune response. For undetermined reasons, ~10% of *Eh* infection sporadically breaches innate mucosal barriers and invades the lamina propria. *Eh* disease pathogenesis is the result of the dynamic interaction of *Eh* with different components of the immune system and the expression of *Eh* virulence factors (Faust and Guillen, 2012; Verkerke et al., 2012; Marie and Petri Jr, 2014; Ghosh et al., 2019; Rosales, 2021). When *Eh* breaches the innate protective mucus barrier (Moncada et al., 2003; Mortimer and Chadee, 2010; Begum et al., 2020a) it comes into direct contact with mucosal epithelial cells and subepithelial macrophages and dendritic cells. Here, NF-κB signaling from epithelial and immune cells plays an indispensable role in shaping the pro-inflammatory landscape during infection (Kammanadiminti and Chadee, 2006; Kammanadiminti et al., 2007; Hou et al., 2010; Begum et al., 2020b). *Eh* components or live *Eh* in direct contact with epithelial cells or macrophages can modulate cellular functions. For example, Caco-2 and T84 human colonic epithelial cells cocultured with differentiated THP-1 macrophages for 24h, followed by stimulation with soluble amebic proteins (SAP) augmented Hsp 27 and 72. In this interaction, Hsp27 played an important role in inhibiting the NF-κB pathway because of its association with the IKK complex while Hsp72 inhibited apoptosis (Kammanadiminti and Chadee, 2006). This may in part, explain why colonic inflammation is not robust in the majority of individuals with intestinal amebiasis. This interaction is not unique to *Eh* as the inhibitory effects of heat shock proteins (Hsp) on NF-κB activation was shown in T-cells (Guzhova et al., 1997). Curiously, the IKK complex seem to be a potential target for Hsp inhibition of the NF-κB pathway (Yoo et al., 2000; Kohn et al., 2002). In another study (Kammanadiminti et al., 2007), *Eh* secreted proteins and SAP induced the expression of the NF-κB dependent cytokine, monocyte chemotactic protein (MCP) from T84, LS174T and Caco-2 epithelial cells. Mechanistically, SAP-induced the phosphorylation of NF-κB p65 subunit and enhanced transcriptional activity that was dependent on phosphatidylinositol 3-kinase (PI3 kinase) **(** **Figure 2** and **Table 1** **)**. Inhibition of PI3 kinase abrogated the activation of Akt, p65, and MCP-1 mRNA induction. What remains unclear from these studies is whether PI3 kinase or Akt directly phosphorylates the p65 subunit in response to ameba components.

**Table 1:** Differential regulation of the NF-κB pathway by protozoan parasites.

Parasite (Disease)	Pathogen component	Target	Result/outcome	Reference
*E. histolytica* (Amebiasis)	SAP	IKK Complex	NF-κB inhibition	(Kammanadiminti and Chadee, 2006)
		Phosphorylation of p65	MCP-1 cytokine induction	(Kammanadiminti et al., 2007)
	Calpain	Degradation of p65, STAT3/5	Cell death	(Kim et al., 2014)
	Gal/GalNAc-lectin	NF-κB and MAPK activation	TLR-2 m-RNA and protein expression	(Kammanadiminti et al., 2004)
	*Eh*CP-A5	IKK activation and IκB phosphorylation	Enhanced pro-inflammatory response	(Hou et al., 2010)
	LPPG	TLR-2 and-4 activation	IL-12p40, TNF-α, IL-10, and IL-8 release	(Maldonado‐Bernal et al., 2005)
	*Eh* genomic DNA	TLR9	NF-κB and MAPK activation	(Ivory et al., 2008)
	Live *Eh*	Cytoskeletal-associated proteins talin, Pyk2 and paxillin	NLRP3 inflammasome activation	(St-Pierre et al., 2017)
*Toxoplasma gondii* (Toxoplasmosis)	TgESAs	Inhibits NF-κBp65 and TLR2/4 activation	Up-regulates IL-10 and TGF-β	(Wang et al., 2017)
	ROP18	p65 degradation	Aborted NF-κB signaling	(Du et al., 2014)
	ROP16	Inhibits STAT3/6 and NF-κB transcription	down-regulates TLR induced cytokines	(Saeij et al., 2007)
	Cathepsin C1	Inhibits p65 phosphorylation	Decrease TNF-α, IL-12, IL-6, IL-8, IL-1 production	(Liu et al., 2019)
	HSP70	Inhibits iNOS and NF-κB	Decrease host parasiticidal mechanism	(Dobbin et al., 2002)
*Plasmodium* (Malaria)	GPI	NF-κB/c-rel	iNOS expression	(Tachado et al., 1996)
	Hemozoin	TLR-9 mediated NF-κB activation	Up-regulates pro-IL-1β and NLRP3 activation	(Coban et al., 2005; Parroche et al., 2007; Baccarella et al., 2013)
*Trypanosoma cruzi* (Chagas)	GPI	Activates TLR2/MyD88, MAPK and NF-κB	Induction of IL-12, TNF-α, and NO	(Campos et al., 2001)
	Cruzipain	Interferes NF-κBp65 signaling	Hinders macrophage activation	(Watanabe Costa et al., 2016)
*Leishmania* (Leishmaniasis)	*L. major* infection	Selectively translocate c-Rel/p50	Induces IL-10 expression	(Guizani-Tabbane et al., 2004)
	gp63	Cleaves NF-κBp65 RelA into p35RelA	Induces expression of MCP-1, MIP-1α, MIP-1β, MIP-2	(Gregory et al., 2008)
	*L. mexicana* infection	Degrades entire NF-κB pathway (p65^RelA^, c-Rel, IκBα, IκBβ, JNK and ERK)	Inhibits IL-12 production	(Cameron et al., 2004)

*In vivo*, the NF-κB p50 subunit played a protective role, as *Eh* challenged C57BL/6 and 129/Sv mice with targeted deletion of the p50 subunit were more susceptible to *Eh* (Cho et al., 2010). A unique mechanism of epithelial cell death was also explored during *Eh* infection (Kim et al., 2014). Curiously, calpain, a calcium-dependent cysteine protease, induced protein degradation of pro-survival transcription factors, including, NF-κB p65, STAT3 and STAT5 that promoted cell death in response to *Eh* (Kim et al., 2014; **Table 1**). *Eh* invasion of the colonic mucosa leads to a pro-inflammatory cytokine burst and recruitment of different immune cells, which includes neutrophils and macrophages to the site of infection (Seydel et al., 1997; Mortimer and Chadee, 2010; Nakada-Tsukui and Nozaki, 2016).

*Eh* deploy an arsenal of virulence factors, which includes amoebapore, galactose/N-acetyl-D-galactosamine (Gal/GalNAc) lectin (Gal-lectin), cysteine proteinases and prostaglandin E_2_ (Moonah et al., 2013; Marie and Petri Jr, 2014). *Eh* Gal-lectin is a major surface molecule that mediates the binding of *Eh* to host cells and to Gal and GalNAc colonic MUC2 mucin glycans (Chadee et al., 1987; Petri et al., 1987). Macrophages are innate immune cells that are instrumental in mounting a robust pro-inflammatory response. Stimulation of macrophages with native Gal-lectin activated NF-κB and MAP kinase signaling pathway that culminated in the induction of TLR-2 mRNA and surface expression (Kammanadiminti et al., 2004; **Figure 2** and **Table 1**). The *Eh* Gal-lectin, a vaccine candidate for amebiasis, induces dendritic cell (DC) maturation and activation *via* MAPK and NF-κB pathway leading to Th1 cytokine production (Ivory and Chadee, 2007). Amongst the different virulence factors, cysteine proteinases play a major role in the pathogenicity of amebiasis (Ankri et al., 1999; Tillack et al., 2006; Meléndez-López et al., 2007). *Eh*CP-A1, *Eh*CP-A2 and *Eh*CP-A5 are highly expressed cysteine proteinases in axenically cultured *Eh* (Bruchhaus et al., 1996; Tillack et al., 2007). The cysteine proteinases repertoire is expressed spatially: *Eh*CP-A1 is confined to intracellular vesicles while *Eh*CP-A5 is expressed on the cell surface, and *Eh*CP-A2 is limited to the inner and outer cell membrane (Jacobs et al., 1998; Que et al., 2002; Meléndez-López et al., 2007). Pro-mature cysteine proteinase 5 (PCP5) is a major virulence factor of *Eh* that is secreted and/or present on the surface of ameba, binds *via* its RGD motif to α_v_β_3_ integrins on colonic cells to trigger NF-κB mediated pro-inflammatory responses (Hou et al., 2010). PCP5-RGD binding to α_v_β_3_ integrins activated integrin-linked kinase (ILK) that mediated the phosphorylation of Akt-473 that subsequently bound and induced IKK activation *via* ubiquitination of NEMO that phosphorylates IκBα triggering pro-inflammatory responses (Hou et al., 2010; **Figure 2**). The Gal-lectin and *Eh*CP-A5 together also play a central role in contact-dependent activation of the NLRP3 inflammasome in macrophages for high output IL-1β secretion (Mortimer et al., 2014; Mortimer et al., 2015). In this interaction, Gal-lectin activates the NF-κB pathway for transcriptional activation of the NLRP3 inflammasome to stimulate TNF-α release (Mortimer et al., 2014). During primary *Eh* infection, macrophage secreted TNF-α has a detrimental outcome leading to increased diarrheal disease. However, naïve macrophages that are primed with TNF-α and IFN-γ produce high levels of nitric oxide (NO) that kills *Eh* (Lin et al., 1994; Seguin et al., 1995; Haque et al., 2007). Several *Eh* components can bind macrophage and epithelial TLR to activate the NF-κB pathway to induce a raging pro-inflammatory response. Mouse macrophages stimulated with *Eh* genomic DNA signaled *via* TLR9 to activate NF-κB and MAPK that was dependent on MyD88 (Ivory et al., 2008; **Figure 2** and **Table 1** **)**. Lipopeptidophosphoglycan (LPPG), a *Eh* associated molecular pattern, activated NF-κB *via* TLR-2 and -4 resulting in the release of IL-12p40, TNF-α, IL-10, and IL-8 from human monocytes (**Table 1**). Mouse macrophages lacking TLR-2 (*TLR-2^-^/^-^*) or deficient in TLR-4 (*TLR-4^d^/^d^*) were unresponsive to LPPG stimulation (Maldonado‐Bernal et al., 2005). *Eh* induced inflammation is characterized by the infiltration of neutrophils, which have been implicated in host defense against amebiasis. Interestingly, *Eh* activates neutrophils to induce extracellular traps that was dependent on the NF-κB pathway (Fonseca et al., 2018). This suggests the if *Eh* can suppress the NF-κB pathway in neutrophils like it does in macrophages, it can ward off potent innate host defenses.

The forgoing discussion elegantly demonstrates that *Eh* and its components can manipulate the NF-κB pathway to elicit a florid pro-inflammatory response that may play a crucial role in *Eh* invasion and shape the outcome of disease. While detailed experimentations have uncovered many unanswered questions during *Eh*-host interaction, there are many questions that still need to be addressed. For instance, which specific NF-κB protein subunits play a regulatory role during *Eh* pathogenesis and what will be the outcome of NF-κB signaling from different cell types upon contact with *Eh*. In this regard we recently (Chadha et al., 2021) uncovered a novel role for inflammatory caspase-1 that intersected NF-κB signaling during *Eh*-macrophage contact. In this interaction, *Eh*-induced caspase-1 activation rapidly degraded cullin-1/5 proteins, a central scaffolding component of multi-subunit E3s ligase that attenuated NF-κB signaling (**Figure 2**) inhibiting TNF-α production. Cullin-1/5 degradation was also observed from colonic epithelial cells following live *Eh* inoculated in proximal colonic loops of mice as a short-term infection model. Cullin-1/5 degradation was dependent on *Eh* surface cysteine proteinases *Eh*CP-A1 and *Eh*CP-A4, but not on *Eh*CP-A5, based on pharmacological inhibition of the cysteine proteinases and *Eh*CP-A5 deficient parasites. These findings highlight that *Eh* suppression of NF-κB signaling induces a predominant NLRP3 dependent IL-1β pro-inflammatory response that may contribute to disease pathogenesis. *Eh* in contact with macrophages is also known to induce the degradation of cytoskeletal-associated proteins talin, Pyk2 and paxillin that activated the NLRP3 inflammasome by an unknown mechanism (St-Pierre et al., 2017; **Table 1**). These findings suggest that *Eh* in contact with host cells at the intercellular junction uses several *Eh* ligands that couples to multiple putative receptors to activate inflammatory caspases and the NF-κB pathway that regulates pro-inflammatory responses. We are now beginning to decipher some of the salient features that regulates *Eh*-host parasite interaction in epithelial cells, macrophages and neutrophils by teasing out defined pathways that may be beneficial to the host and/or parasite in disease pathogenesis.

## NF-κB Pathway Modulation During *Toxoplasma gondii* Infection

Unlike extracellular *Eh*, intracellular protozoan parasites have devised unique ways to modulate the innate immune response *via* inside-out signaling by manipulating the NF-κB pathway. *T. gondii*, the causative agent of toxoplasmosis, is an obligatory intracellular protozoan parasite that can infect all nucleated cells of warm-blooded animals (Hou et al., 2019; Li et al., 2019; de Faria Junior et al., 2021) including wild, domesticated and companion animals (Dubey et al., 2012). It infects about one-third of the world’s human population (Sasai et al., 2018). Infection in immunocompromised individuals often leads to symptomatic and lethal toxoplasmosis (Tenter et al., 2000). Humans and other animals become infected due to consumption of under-cooked meat of infected animals or by ingesting water or food contaminated with oocysts (Jones et al., 2005; Dubey and Jones, 2008). *T. gondii* has three infectious stages known as tachyzoite, bradyzoite and sporozoites (within oocysts) (Dubey et al., 1998). Mouse models identified three different strains of *T. gondii* called type I, type II, and type III with different virulence factors. Amongst the three strains, type I is the most virulent strain, while type II and type III are avirulent (Howe et al., 1996; Mordue et al., 2001).

To counteract the host immune responses, *Toxoplasma* deploys multiple strategies to subvert the NF-κB signaling pathway. Infection of bone marrow-macrophages with RH tachyzoites (RH strain of *T. gondii*, which is a type I representative strain) repressed NF-κB activation by inhibiting nuclear localization of p65 or c-Rel, while *in-vivo* infection activated the NF-κB pathway (Shapira et al., 2002). While the pathogen displays a repertoire of virulence factors, some play a crucial role in establishing the infection *via* immunomodulation of different immune cells. *T. gondii* excretory/secretory antigens (TgESAs), a virulence factor, inhibited nuclear translocation of NF-κBp65 and TLR-2 and -4 activation from LPS-stimulated Ana-1 murine macrophage that upregulated the anti-inflammatory cytokines IL-10 and TGF-β and downregulated the pro-inflammatory cytokines TNF-α and IL-1β (Wang et al., 2017; **Figure 2** and **Table 1**). One of the strategies used by the parasite to subvert immune responses, is degradation of host proteins and transcription factors essential for regulating the immune response. *T. gondii* releases its protein into the host from organelles called dense granules and rhoptries (ROPs), thus manipulating host cell and their transcriptional responses (Lima and Lodoen, 2019; Tuladhar et al., 2019). ROP18, an effector of type I strains, is a serine/threonine kinase that modulates the phosphorylation of host proteins to circumvent cell signaling pathways. Surprisingly, ROP18 induced the phosphorylation of p65 at Ser-468 that led to ubiquitin-dependent degradation of p65 culminating in aborted NF-κB signaling, thus conferring a survival advantage (Du et al., 2014; **Figure 2** and **Table 1**). Another protein ROP16, a putative protein kinase, suppressed IL-12 responses in infected macrophages stimulated with TLR agonist (Saeij et al., 2007) and inhibited NF-κB transcriptional activity (Rosowski et al., 2011), possibly due to the activation of STAT3/6 (Saeij et al., 2007) that downregulated TLR-induced cytokine production (**Table 1**). In contrast, *T. gondii* strains that express dense granule protein GRA15 directly activates NF-κB through a MyD88-independent mechanism (Melo et al., 2011). Recently (Liu et al., 2019), *T. gondii* cathepsin C1 (CPC1), a member of the GRA (dense granule) protein family, was shown to inhibit the phosphorylation of p65 subsequently leading to decreased production of pro-inflammatory cytokines TNF-α, IL-12, IL-6, IL-8 and IL-1 (**Figure 2** and **Table 1**). CPC1 inhibited NF-κB activation through positive regulation of HIF (hypoxia-inducible factor)-1α/EPO (erythropoietin) axis (Liu et al., 2019). While several studies have indicated the involvement of the NF-κB pathway during *T. gondii* infection, it seems to be cell-specific regulation. Heat shock protein 70 (HSP70) of *T. gondii* inhibited parasiticidal activity by inhibiting iNOS, and NF-κB activation from RAW 264.7 and splenocytes, respectively (Dobbin et al., 2002; **Figure 2** and **Table 1**). Surprisingly, *T. gondii* infected macrophage up-regulated the phosphorylation and degradation of IκB and blocked the translocation of NF-κB by inhibiting the phosphorylation of p65/RelA (Shapira et al., 2005) leading to aborted pro-inflammatory cytokine production (Butcher et al., 2001; Shapira et al., 2002). While these results are well documented in murine macrophages it is still debatable if a similar mechanism occurs in murine fibroblasts (Shapira et al., 2002; Molestina et al., 2003). LPS induced IL-1β production inhibition from primary human neutrophils following type 1 strain infection was associated with inhibition of NF-κB. Although neutrophils infected with *T. gondii* aborted NF-κB signaling *via* reduced IκBα degradation and p65/RelA phosphorylation, it also showed marked reduction in transcripts for NLRP3 inflammasome sensor and IL-1β (Lima et al., 2018). To assess the importance of NF-κB during the infection, mice deficient in specific genes belonging to the NF-κB pathway have been assessed. Mice lacking RelB succumb to acute infection, due to inability to produce IFN-γ indicating an indispensable role of RelB in conferring resistance to *T. gondii* infection (Caamaño et al., 1999). During chronic infection, *NF-κB_2_^-^/^-^* mice have higher mortality when compared to wild-type (WT) mice due to global T-cell loss and apoptosis (Franzoso et al., 1998). Previous studies have shown altered microRNA expression profile by Apicomplexan parasites (Deng et al., 2004; McDonald et al., 2013; Hou et al., 2019) indicating the involvement of microRNA during infections. *T. gondii* infection perturbed the signaling pathways responsible for generating host defense responses (Hakimi and Ménard, 2010) by modulating the expression of host microRNAs, which contributes to efficient parasite replication (Cong et al., 2017). In agreement with these observations, *T. gondii* attenuated the NF-κB pathway by inducing miR-146a in the host (Taganov et al., 2006). STAT3 and NF-κB activation in response to *T. gondii* up-regulated the expression of miRNAs miR-125b-2, miR-30c-1, miR-17-92 and miR-23b-27b-24-1 (Cai et al., 2013). Taken together, these observations suggest that *T. gondii* exploits the NF-κB pathway for successful replication and to evade cell mediated immunity.

## Role of NF-κB in Other Protozoan Parasites

As NF-κB signaling is crucial for mounting an immediate immune response against invading pathogens, its manipulation has been described at multiple levels in response to several protozoan parasites. *Plasmodium* is the etiologic agent of the disease malaria. According to the WHO report 2015, it infects over 200 million people annually and kills over 500,000 patients a year (World Health Organization (WHO, 2016). Glycosylphosphatidylinositol (GPI) of plasmodium activates macrophages and endothelial cells inducible NO synthase expression that involves NF-κB/c-rel (Tachado et al., 1996; **Table 1** **)**. Hemozoin, a malarial pigment, binds to TLR9 and activates NF-κB and the NLRP3 inflammasome to increase the levels of pro-IL-1β (Coban et al., 2005; Parroche et al., 2007; Baccarella et al., 2013; **Figure 2** and **Table 1** **)**. A recent study (Toda et al., 2020) demonstrated a role for plasma-derived extracellular vesicles (EVs) from *P. vivax* patients (PvEVs) that activated NF-κB translocation from human spleen fibroblasts (hSFs), which up-regulated the levels of ICAM-1 that resulted in specific adhesion properties of reticulocytes (from infected patients) to hSFs (Toda et al., 2020). *Trypanosoma cruzi* the causative agent of Chagas disease, infects over 5 million people across the globe and kills thousands of people each year (Pérez-Molina and Molina, 2018). Cytokines released by immune cells play a decisive role in disease pathogenesis and invasion by infectious agent. The Y strain of *T. cruzi* was shown to activate NF-κB *via* the TNF pathway that increased invasion of non-professional phagocytic epithelial cells demonstrating a negative role for NF-κB activation favoring the parasite (Pinto et al., 2011). *T. cruzi* GPI, a pathogen-associated molecular pattern, is recognized by TLR-2, which stimulates the TLR-2/Myd88 pathway, MAPK and NF-κB transcription factor activation (Campos et al., 2001; Takeda and Akira, 2005; **Table 1**). In contrast, cruzipain, a *T. cruzi* secreted lysosomal peptidase, hindered macrophage activation during the initial stages of infection by interfering with NF-κBp65 mediated signaling (Watanabe Costa et al., 2016; **Figure 2** and **Table 1**). Leishmaniasis, caused by multiple *Leishmania* species, is responsible for an estimated 12 million infections across the globe and thousands of deaths per year (Lozano et al., 2012; Vos et al., 2016). Different *Leishmania* species differentially regulate the NF-κB pathway. For instance, *L. major* infected monocytes (primary and PMA-differentiated U937 cells) inhibited nuclear localization of p65^RelA^/p50 heterodimers, however, it selectively promoted the translocation of c-Rel/p50 heterodimers, which induced the anti-inflammatory cytokine, IL-10 (Guizani-Tabbane et al., 2004; **Figure 2** and **Table 1**). Infection of murine-BMDM with *L. mexicana* amastigotes degraded the entire NF-κB pathway; degradation of p65^RelA^, c-Rel, the upstream kinases JNK and ERK and the inhibitors IκBα and IκBβ (Cameron et al., 2004; **Figure 2** and **Table 1**). In contrast, another group showed a novel subversion mechanism, wherein Leishmania protease, gp63, *in vitro* cleaved NF-κB p65RelA that resulted in a fragment p35RelA that dimerized with p50, which induced gene expression of the chemokines MCP-1, MIP-1α, MIP-1β and MIP-2 (Gregory et al., 2008; **Table 1**). A comprehensive view of the regulation of the NF-κB pathway by protozoan parasites is listed in **Table 1** and **Figure 2** summarizes the differential regulation of the NF- κB pathway by different protozoan parasites and their virulence factors.

## Conclusion and Future Direction

The immune system is armored with multiple receptors, which are recognized by invading pathogens culminating in gene expression associated with the development of an immune response. Parasite interaction with the innate immune response involves coupling though multiple receptors that activates the NF-κB pathway. From an evolution point of view, multiple strategies reflect the selective pressure this pathway has imposed on different pathogens, while in turn evolution of different pathogens have led to the diversification of this pathway (Tato and Hunter, 2002). From the forgoing discussion it is apparent that parasites deploy multiple ways to circumvent signaling *via* the NF-κB pathway. However, we know very little on the diverse array of parasite molecules and/or downstream signaling involved in NF-κB activation and inhibition by extracellular and intracellular protozoan parasites. NF-κB pathway diversification involves different protein subunits that form different hetero/homodimers (Gilmore, 2006). Intriguingly, different combination and permutation of these dimers have different functional consequence on gene expression responsible for immune activation/inhibition. At present, we still do not know which specific homo/heterodimer subunits are formed during contact and/or invasion by parasites, and what would be the functional consequence. The question that is still baffling and needs attention is, whether NF-κB activation by different parasites favors the host or the pathogen or both. The dichotomy in NF-κB activation and inhibition observed by extracellular and intracellular parasites, in part, may answer why intracellular parasites inhibit this pathway, while extracellular parasites activates it. It is essential to understand which specific NF-κB subunit play an indispensable role during parasitic infection and how different receptor sense these parasites in a cell-type specific manner. Understanding these pathways could provide a better appreciation on the complexity of the disease and thus, help to develop better therapeutic approach for parasitic infections.

## Author Contributions

AC and KC conceived the review topic and wrote the manuscript. All authors contributed to the article and approved the submitted version.

## Funding

This work was funded by a Discovery Grant (RGPIN-2019-04136) from the Natural Sciences and Engineering Research Council of Canada and a project grant from the Canadian Institutes of Health Research (PJT-407276) awarded to KC.

## Conflict of Interest

The authors declare that the research was conducted in the absence of any commercial or financial relationships that could be construed as a potential conflict of interest.

## Publisher’s Note

All claims expressed in this article are solely those of the authors and do not necessarily represent those of their affiliated organizations, or those of the publisher, the editors and the reviewers. Any product that may be evaluated in this article, or claim that may be made by its manufacturer, is not guaranteed or endorsed by the publisher.
